# Cell numbers in the reflected blade of CA3 and their relation to other hippocampal principal cell populations across seven species

**DOI:** 10.3389/fnana.2022.1070035

**Published:** 2023-01-04

**Authors:** Jovana Maliković, Irmgard Amrein, Lorenzo Vinciguerra, Dušan Lalošević, David P. Wolfer, Lutz Slomianka

**Affiliations:** ^1^Division of Functional Neuroanatomy, Institute of Anatomy, University of Zürich, Zürich, Switzerland; ^2^Department of Health Sciences and Technology, ETH Zürich, Zürich, Switzerland; ^3^Natural History Museum, St. Gallen, Switzerland; ^4^Pasteur Institute of Novi Sad, Novi Sad, Serbia; ^5^Department of Histology and Embryology, Faculty of Medicine, University of Novi Sad, Novi Sad, Serbia

**Keywords:** hippocampus, dentate gyrus, calretinin, optical fractionator, comparative neuroanatomy

## Abstract

The hippocampus of many mammals contains a histoarchitectural region that is not present in laboratory mice and rats—the reflected blade of the CA3 pyramidal cell layer. Pyramidal cells of the reflected blade do not extend dendrites into the hippocampal molecular layer, and recent evidence indicates that they, like the proximal CA3 pyramids in laboratory rats and mice, partially integrate functionally with the dentate circuitry in pattern separation. Quantitative assessments of phylogenetic or disease-related changes in the hippocampal structure and function treat the reflected blade heterogeneously. Depending on the ease with which it can be differentiated, it is either assigned to the dentate hilus or to the remainder of CA3. Here, we investigate the impact that the differential assignment of reflected blade neurons may have on the outcomes of quantitative comparisons. We find it to be massive. If reflected blade neurons are treated as a separate entity or pooled with dentate hilar cells, the quantitative makeup of hippocampal cell populations can differentiate between species in a taxonomically sensible way. Assigning reflected blade neurons to CA3 greatly diminishes the differentiating power of all hippocampal principal cell populations, which may point towards a quantitative hippocampal archetype. A heterogeneous assignment results in a differentiation pattern with little taxonomic semblance. The outcomes point towards the reflected blade as either a major potential player in hippocampal functional and structural differentiation or a region that may have cloaked that hippocampi are more similarly organized across species than generally believed.

## Introduction

**Figure 1:**
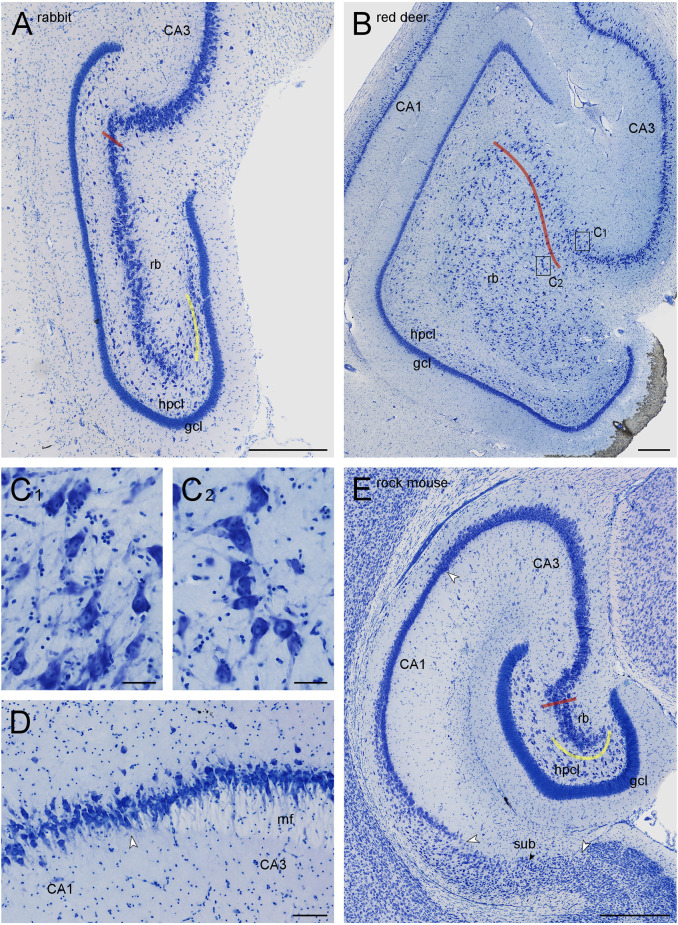
Dentate gyri of the New Zealand white rabbit **(A)** and the red deer **(B,C)**, distal tip of the mossy fiber zone in boar **(D)**, and hippocampal formation of the Namaqua rock mouse **(E)**. Red lines mark the border of the CA3 reflected blade (rb) towards the remainder of CA3. Yellow lines mark the boundary between the reflected blade and the hilar polymorphic cell layer (hpcl) in species in which they partially merge. Rectangles in **(B)** labeled **(C1,C2)** mark the positions of the high magnification images **(C1,C2)**. Large white arrowheads in **(D)** mark subregional boundaries. The small black arrow in **(E)** marks the tentative boundary between the proximal subiculum (or prosubiculum) and the distal subiculum. The apical dendrites of CA3 pyramidal cells consistently point towards the superficial layers of CA3 **(C1)**, whereas the apical dendrites of CA3 reflected blade pyramids **(C2)** point towards the granule cell layer, even though the hippocampal layers would be much closer. At the transition from CA3 to CA1 **(D)**, a background staining of the mossy fiber zone (mf) is lighter than that of the adjacent stratum radiatum, clearly marking the border of CA3 in most sections of all species. Scale bars: **(A,B,** and **D)** 0.5 mm; **(C1)** and **(C2)** 50 μm; **(C3)** 100 μm.

The hippocampus of many species, including humans and other primates, contains an easily recognizable histoarchitectural region that is not present in laboratory rats and mice—the reflected blade of CA3. Briefly, as the CA3 pyramidal cell layer inserts into the curvature of the dentate gyrus, the layer first turns towards the suprapyramidal blade of the dentate granule cell layer. Next, it bends back sharply (reflects) to run for some distance more or less parallel to the dentate layers (Figure 1A) towards the infrapyramidal blade of the dentate layers. In some species, the CA3 reflected blade neurons are separated from the hilar polymorphic cell layer by a continuation of the stratum radiatum. In others, the two layers merge at least for some distance along their course. Among the species that are still used in the laboratory, the CA3 reflected blade is present in guinea pig (Geneser-Jensen, 1972, 1973) and rabbit (Geneser, 1987). The nomenclature of the reflected blade of the CA3 pyramidal cell layer is bewilderingly inconsistent, both with regard to the term used to name the reflected blade and with regard to the larger anatomical unit to which it may belong. Lorente de Nó (1934), who coined the term reflected blade, actually named it the *first* reflected blade; the second reflected blade being the hilar polymorphic cell layer. The reflected blade has been termed CA4 (Rosene and van Hoesen, 1987; Nitsch and Ohm, 1995; Harding et al., 1998; Ding, 2015), but this term has also been used for a combined reflected blade and hilar polymorphic cell layer (Geneser, 1987; Lorente de Nó, 1934; Schwerdtfeger, 1984) or just the hilar polymorphic cell layer (van Groen and Wyss, 1988). The reflected blade has also been pooled with CA3 (Amaral and Insausti, 1990; Kondo et al., 2009) or was suggested to represent CA3c (Seress et al., 2008) of laboratory mice and rats. Finally, it was considered a distinct hilar population of CA3 termed CA3h or CA3 end (Lim et al., 1997a; Slomianka et al., 2013; Ding and van Hoesen, 2015; Blackstad et al., 2022).

Here, we return to the original term reflected blade because it is unequivocal and concisely descriptive.

The heterogeneous nomenclature may reflect an uncertainty about the anatomical and functional affiliations of this part of the CA3 pyramidal cell layer. Beyond its special topography and the adage that form follows function, there is only sparse evidence that pyramidal cells located in the reflected blade of CA3 show distinct morphological and physiological characteristics. In pig, sheep, and rhesus macaques, typical CA3 pyramidal cells are supplemented by non-apical and dentate CA3 pyramidal cells (Buckmaster and Amaral, 2001; Buckmaster, 2005; Blackstad et al., 2022). The non-apical pyramidal cells do not send dendrites into apical layers of CA3, while the dentate pyramidal cells (and also reflected blade interneurons) extend dendrites into the dentate polymorphic and granule cell layers to enter and branch in the dentate molecular layer cells (Pitkänen and Amaral, 1993; Buckmaster and Amaral, 2001). Both non-apical and dentate CA3 pyramidal cells extend axons into the dentate layers and may receive axons from dentate mossy cells (Buckmaster and Amaral, 2001). In the guinea pig reflected blade, a population of non-bursting CA3 pyramidal neurons, with multiple apical dendrites arising directly from the soma, is more responsive to stimulation than non-bursting cells found elsewhere in CA3 (Bilkey and Schwartzkroin, 1990).

In addition to the sparse reports on characteristics specific to the reflected blade, the most proximal part of the CA3 pyramidal cell layer in laboratory mice and rats may shed some additional light on the function of reflected blade pyramids. While the dendrites of proximal CA3 pyramids avoid the dentate layers (Amaral, 1978; Buckmaster et al., 1993), non-apical pyramids are also present, and EPSPs and IPSPs following molecular layer stimulation are lower than in other parts of CA3 (Sun et al., 2017). Proximal CA3 cells also project to the dentate layers (Ishizuka et al., 1990; Li et al., 1994; Scharfman, 1994, 1996, 2007; Kneisler and Dingledine, 1995), and modeling studies suggest that this back-projection may facilitate pattern separation (Myers and Scharfman, 2011; Petrantonakis and Poirazi, 2015). Functional activation and arc expression are indeed strongest in proximal CA3 in a pattern separation task, while pattern completion preferentially activates distal CA3 (Marrone et al., 2014; Lee et al., 2015; Lu et al., 2015; Sun et al., 2017). Autoassociative connections are relatively sparse in proximal CA3 (Sun et al., 2017), which anatomically reflects the shift from pattern completion to pattern separation. Not surprisingly, functional deficits following proximal CA3 lesions resemble dentate lesions (Hunsaker et al., 2008), and, interestingly, hyperactivity restricted to proximal CA3 is involved in age-related memory impairments (Lee et al., 2021). The tight functional integration of the dentate and proximal CA3 that some of these studies argue for (GoodSmith et al., 2019; Lee et al., 2020) may be further developed in species with a reflected blade, an argument that has been made before for the primate hippocampus (Lim et al., 1997a).

Quantitative morphological studies are less ambiguous with regard to the assignment of the reflected blade, albeit more for the sake of convenience/feasibility than because of functional considerations. Because the reflected blade can be difficult to separate from the hilar polymorphic cell layer, it is usually considered to be part of a hilar cell population that comprises both CA3 reflected blade cells and hilar polymorphic cells (West and Gundersen, 1990; Holm and West, 1994; Harding et al., 1998; Keuker et al., 2003; Slomianka et al., 2013; Rogers Flattery et al., 2020; Schilder et al., 2020). In a study of the comparative quantitative anatomy of the hippocampal principal cell population (van Dijk et al., 2016a), we also used this assignment and found relative hilar and CA3 cell populations sizes to be one of the major differentiators between species. Even though the functional integration of proximal CA3 and the dentate gyrus does provide an argument for this assignment, one cannot but wonder how quantitative relations would be impacted if reflected blade cells were considered as a separate cell population or if they were assigned to CA3. To answer this question, we present in this study a quantitative analysis of hippocampal principal cell numbers in seven species, in which the cells of the reflected blade of CA3 and the hilar polymorphic cells could be reliably distinguished—Namaqua rock mouse, Dunkin Hartley guinea pig, New Zealand white rabbit, golden jackal, wild boar, European roe deer, and red deer. Furthermore, we analyze these seven species with respect to quantitative data from the literature for humans, rhesus monkey, and C57BL6 mice.

## Materials and Methods

### Animals and tissue preparation

Brains of Dunkin Hartley guinea pigs, New Zealand white rabbits, golden jackals, wild boar, European roe deer, and red deer were processed for this study (Table 1). All brains were collected *post mortem* from animals provided to us either by local hunters and game wardens in agreement with federal game law and the cantonal regulations for species population control or received through collaborations with the Laboratory Animal Services Centre, University of Zurich, Switzerland, and Idorsia Pharmaceuticals Ltd, Allschwil, Switzerland. The age of wild animals were estimated by the hunters or game wardens. Sections of Namaqua rock mice were available from our previous work (Cavegn et al., 2013 and Table 1). Additional cell numbers for humans, rhesus macaque, and C57B6 were available from published data (Table 1).

**Table 1:** List of species, their latin names, orders, sources, and permits or references.

**Common name**	**Latin name**	**Order**	**Origin/Source**	**Permit/Reference**
Namaqua rock mouse	*Micaelamys namaquensis*	Rodentia	Limpopo province South Africa	Cavegn et al. (2013)
Dunkin Hartley guinea pig HsdDhl:DH	*Cavia porcellus*	Rodentia	Idorsia (Envigo, The Netherlands)	Cantonal Veterinary Office #BL170/30695
C57BL6	*Mus musculus domesticus*	Rodentia	Literature	Fabricius et al. (2008) and van Dijk et al. (2016b)
New Zealand white rabbit NZW:Hsd1lf	*Oryctolagus cuniculus domesticus*	Lagomorpha	LASC UZH, Zurich, Switzerland	Cantonal Veterinary Office #ZH195/2013
Golden jackal	*Canis aureus*	Carnivora	Irig, Serbia	Law on game and hunting
wild boar	*Sus scrofa*	Artiodactyla	Canton of Zurich, Switzerland	Federal game law, permits to authorized hunters
European roe deer	*Capreolus capreolus*	Artiodactyla	Canton St. Gallen, Switzerland	Federal game law, permits to authorized hunters
red deer	*Cervus elaphus*	Artiodactyla	Canton St. Gallen, Switzerland	Federal game law, permits to authorized hunters
Rhesus macaque	*Macaca mulatta*	Primates	Literature	Keuker et al. (2003)
Human	*Homo sapiens*	Primates	Literature	West and Gundersen (1990); West (1993); and Simic et al. (1997)

Brains were removed from cranial cavities within ~10 min (guinea pig, rabbit) to ~4 h (jackal, wild boar, roe deer, red deer) after death, weighed and immersion fixed in three changes of 4% phosphate-buffered paraformaldehyde containing 15% picric acid for a total of 7 days. Small brains were processed *in toto* (rock mouse, guinea pig, and rabbit). The hippocampi and entorhinal cortex of large brains were dissected (red deer, roe deer, boar, and jackal). Thereafter, one hemisphere/hippocampus of each animal was cryoprotected in 30% sucrose, frozen with CO_2,_ and stored at −80°C until further processing, while the second hemisphere/hippocampus was embedded in 2-hydroxyethyl-methacrylate (2-HEMA, see below).

### Immunohistochemistry

To aid in the identification of the hippocampal cell populations, we stained sections for calretinin, which marks dentate mossy cells in laboratory rodents (Kotti et al., 1996; Blasco-Ibáñez and Freund, 1997; Fujise et al., 1998; Murakawa and Kosaka, 1999, 2001), parvalbumin and calbindin, which show regionally specific expression profiles in many species (Rami et al., 1987; Holm et al., 1990; de Jong et al., 1996; Hof et al., 1996; Slomianka et al., 2013; Pillay et al., 2021). Features that have been described repeatedly for several species were used as additional confirmation of the interregional border visible in the Giemsa-stained sections. Specific features that we used are mentioned in the section on the definitions of cell populations. As they have been described in multiple species already, they are not illustrated for calbindin and parvalbumin.

Tissue blocks were cut at 40 μm on a sliding microtome (Zeiss, Germany). Both coronal and horizontal sections were prepared from the hemisphere processed *in toto*. Large, dissected hippocampi were split in the middle, and the two blocks were cut along the long axis of the hippocampus. Sections were collected in series and stored in cryoprotectant at −20°C until further processing. Immunohistochemistry was performed on free-floating serial sections spanning the entire hippocampus. For all antigens, mouse brain sections were processed in the same batch. None of the antibodies stained structures that, based on concurrently processed mouse sections and published distributions of the antigens, were regarded unspecific.

For calbindin and calretinin, microwave epitope retrieval was performed in citric buffer (Sigma Aldrich 1:10 in dH_2_O) for three heating cycles. Afterwards, sections were incubated for 1 h in antibody diluent (2% normal goat (calbindin and calretinin) or horse (parvalbumin) serum and 0.2% Triton in Tris-Triton). Sections were then incubated overnight at room temperature (rabbit anti-calretinin, Swant, Lot 1893-0114—red deer, roe deer, wild boar, guinea pig: 1:1,000, jackal: 1:800, rabbit, Namaqua rock mouse: 1:500/rabbit anti-calbindin, Swant, Lot 9.03—all species: 1:5,000/mouse anti-parvalbumin, Sigma Aldrich, Lot100M4797—red deer, roe deer: 1:200; wild boar, rabbit: 1:5,000; jackal: 1:2,000; guinea pig, Namaqua rock mouse: 1:1,000) in antibody diluent. After incubation, sections were washed in TBS and incubated in secondary antibody (goat anti rabbit, 1:300, Vector Lot X11041 or horse anti-mouse, 1:300, Vector, Lot ZF0521) for 40 min at room temperature. The sections were then DAB stained, mounted on gelatinized slides, dried, dehydrated, cleared and cover-slipped.

### Golgi-Cox staining

After decapitation, the brains of two white rabbits were briefly rinsed with distilled water, and the hippocampi were dissected. Golgi-Cox staining was performed using FD Rapid GolgiStain^TM^ Kit (FD NeuroTechnologies, INC., Belgium). Hippocampi were transferred to plastic tubes with premixed Golgi-Cox impregnation solution and stored in the dark at room temperature. The impregnation solution was changed after 24 h, and hippocampi were impregnated for two additional weeks. Samples were then transferred into the kits cryo-protective solution for 72 h. Tissue blocks were cut perpendicular to the hippocampal long axis, and 150 μm thick frozen sections were cut on a sliding microtome. Sections were placed on gelatin-coated slides, toned according to kit’s protocol, dehydrated, cleared, and cover-slipped with Eukitt.

### Stereology

Dissected hemispheres/hippocampi and entorhinal cortices were washed in PBS and dehydrated in ascending alcohols over 2 days. Pre-infiltration with a 1:1 solution of 100% alcohol and 2-HEMA (Technovit 7100, Heraeus Kulzer GmbH, Wehrheim/Ts, Germany) overnight was followed by three changes of 2-HEMA alone for a total period of 3 weeks. To facilitate the definition of interregional boundaries, the orientation of the sections was selected to cut the temporal hippocampus along its long axis. Rock mouse and guinea pig hemispheres were sectioned horizontally. Rabbit, jackal, wild boar, roe deer, and red deer were sectioned coronally. Sections were cut at 20 μm, mounted, and dried at 60°C for 1 h. Sections were Giemsa stained following the protocol of Iñiguez et al. (1985), cleared and cover-slipped.

Neuron number estimates were performed with StereoInvestigator 10 Software (MBF Bioscience, Williston, VT, USA). To design preliminary sampling schemes (Slomianka, 2021), area estimates of the regions of interest were performed using a point counting approach in one animal of each species. Total neuron numbers were estimated from counts using the optical fractionator (West et al., 1991). Sections were sampled with 10 μm high disectors and 2 μm top guard zones. Section thickness was estimated at every 5th sampling site. Counts were performed using a ×63 oil immersion lens (NA 1.4). All sampling parameters and cell counts are listed in Table 2. Total number estimates based on number-weighted section thickness (Dorph-Petersen et al., 2001), mean coefficients of error (CEs, Gundersen et al., 1999) of the individual estimates for a conservative *m* = 0 and CE^2^/CV^2^ ratios are listed in Table 3. Differences between number estimate based on number-weighted section thickness or mean section thickness were, across all cell populations, less than 1%. Considering that both CE^2^ and CV^2^ are estimates based on relatively small samples, large deviations of the CE^2^/CV^2^ from the target value of 0.5 (roe deer hilar cells, red deer CA3 pyramidal cells, or wild boar granule cells) can occur but did not occur more often than can be expected. Most deviations were related to unusually small group variances, while mean CE estimates were on target, at or below 0.1.

**Table 2:** Sampling parameters of the optical fractionator cell counts, the number of sections that were analyzed, and the raw cell counts that were obtained from these sections.

	**Namaqua rock mouse**	**Guinea pig**	**White rabbit**	**Golden jackal**	**Wild boar**	**European roe deer**	**Red deer**
Section sampling fraction (reflected blade)	1/12th (1/6th)	1/12th	1/30th	1/40th (1/20th)	1/50th	1/60th	1/100th
Range of section numbers	18–22	16–36	15–22	16–22	13–19	15–16	19–22
**Granule cells**							
Counting frame, μm	15 × 15	15 × 15	15 × 15	15 × 15	20 × 20	20 × 20	20 × 20
Step size, μm	220	220	200	340	380	400	400
Counts, mean (SD)	312 (58)	239 (89)	359 (191)	147 (26)	235 (181)	282 (102)	155 (37)
**Hilar cells**							
Counting frame, μm	60 × 60	60 × 60	60 × 60	70 × 70	90 × 90	90 × 90	90 × 90
Step size, μm	200	220	270	340	380	600	400
Counts, mean (SD)	129 (27)	264 (89)	121 (31)	116 (23)	177 (71)	94 (7)	123 (51)
**CA3 reflected blade**							
Counting frame, μm	60 × 60	50 × 50	40 × 40	70 × 70	70 × 70	90 × 90	70 × 70
Step size, μm	200	220	230	190	380	600	450
Counts, mean (SD)	163 (60)	156 (65)	124 (37)	305 (92)	166 (75)	144 (34)	96 (35)
**CA3 pyramidal cells**							
Counting frame, μm	40 × 40	40 × 40	35 × 35	60 × 60	60 × 60	70 × 70	70 × 70
Step size, μm	250	220	200	420	380	600	450
Counts, mean (SD)	308 (78)	350 (87)	245 (81)	235 (11)	225 (75)	135 (11)	147 (60)
**CA1 pyramidal cells**							
Counting frame, μm	40 × 40	30 × 30	20 × 20	20 × 20	45 × 45	45 × 45	50 × 50
Step size, μm	320	220	220	560	470	600	450
Counts, mean (SD)	301 (41)	432 (99)	284 (56)	148 (6)	246 (98)	174 (28)	168 (108)
**Subicular cells**							
Counting frame, μm	40 × 40	40 × 40	50 × 50	50 × 50	70 × 70	70 × 70	60 × 60
Step size, μm	320	220	370	400	470	600	600
Counts, mean (SD)	129 (22)	312 (100)	166 (56)	165 (29)	212 (89)	169 (44)	127 (31)

**Table 3:** Total cell number estimates for the six cell populations that were studied, mean relative variability of estimates due to the estimation procedure (mean CE), and contribution of variability due to the estimation procedure to the observed group variance (CE^2^/CV^2^).

	**Namaqua rock mouse**	**Guinea pig**	**White rabbit**	**Golden jackal**	**Wild boar**	**European roe deer**	**Red deer**
*n* (by sex)	6 (f:3; m:3)	6 (m:6)	6 (f:6)	6 (f:3; m:3)	6 (f:2; m:4)	6 (f:3; m:3)	4 (f:2; m:2)
**Granule cells**							
mean	1,453,613	2,395,757	3,633,053	5,618,709	9,774,480	11,747,368	14,877,360
SD	176,977	472,591	501,175	1,280,617	7,424,547	3,578,945	5,571,669
mean CE	0.08	0.09	0.07	0.09	0.08	0.09	0.1
CE^2^/CV^2^	0.43	0.21	0.26	0.16	0.01	0.09	0.07
**Hilar cells**							
mean	39,599	154,585	168,059	219,237	381,697	443,610	839,590
SD	5,698	35,606	26,343	30,874	155,755	15,788	202,272
mean CE	0.1	0.08	0.1	0.1	0.12	0.11	0.12
CE^2^/CV^2^	0.48	0.12	0.41	0.50	0.09	9.55	0.25
**CA3 reflected blade**							
mean	25,481	151,041	290,789	106,217	651,072	776,156	1,236,229
SD	7,806	31,265	77,215	19,218	261,971	138,933	157,869
mean CE	0.08	0.17	0.12	0.09	0.1	0.1	0.09
CE^2^/CV^2^	0.07	0.67	0.20	0.25	0.06	0.31	0.50
**CA3 pyramidal cells (without reflected blade)**							
mean	314,836	500,786	606,882	902,420	1,061,602	1,140,473	1,820,602
SD	65,222	52,937	109,846	94,714	251,170	173,963	118,638
mean CE	0.08	0.07	0.08	0.08	0.08	0.09	0.10
CE^2^/CV^2^	0.15	0.44	0.20	0.58	0.11	0.35	2.55
**CA1 pyramidal cells**							
mean	507,266	1,023,069	2,362,651	1,472,073	2,942,298	3,314,978	5,706,319
SD	49,494	195,018	393,902	207,432	1,286,660	617,870	1,625,459
mean CE	0.09	0.09	0.06	0.09	0.08	0.08	0.1
CE^2^/CV^2^	0.85	0.22	0.13	0.41	0.03	0.18	0.12
**Subicular cells**							
mean	229,809	397,919	669,099	927,230	1,096,495	1,355,339	2,927,732
SD	34,261	38,900	75,806	124,011	301,534	356,599	772,318
mean CE	0.09	0.08	0.09	0.08	0.09	0.09	0.11
CE^2^/CV^2^	0.36	0.67	0.63	0.36	0.11	0.12	0.16

### Definitions of the cell populations

The definitions given below correspond to the ones most commonly used in the current literature. They use the terms defined by Lorente de Nó (1934) as they can be applied to the cell population and regions defined by Cajal (1968). They also correspond to the definitions of populations that have been investigated in the quantitative studies that we obtained data from and/or compare our results with. Subfields or sublayers have been defined within each of the regions and, they have sometimes been elevated to independent regions—most notably CA2 and the prosubiculum. We briefly comment on these two regions below, although providing quantitative data is beyond both the scope and possibilities of this work.

**Figure 2:**
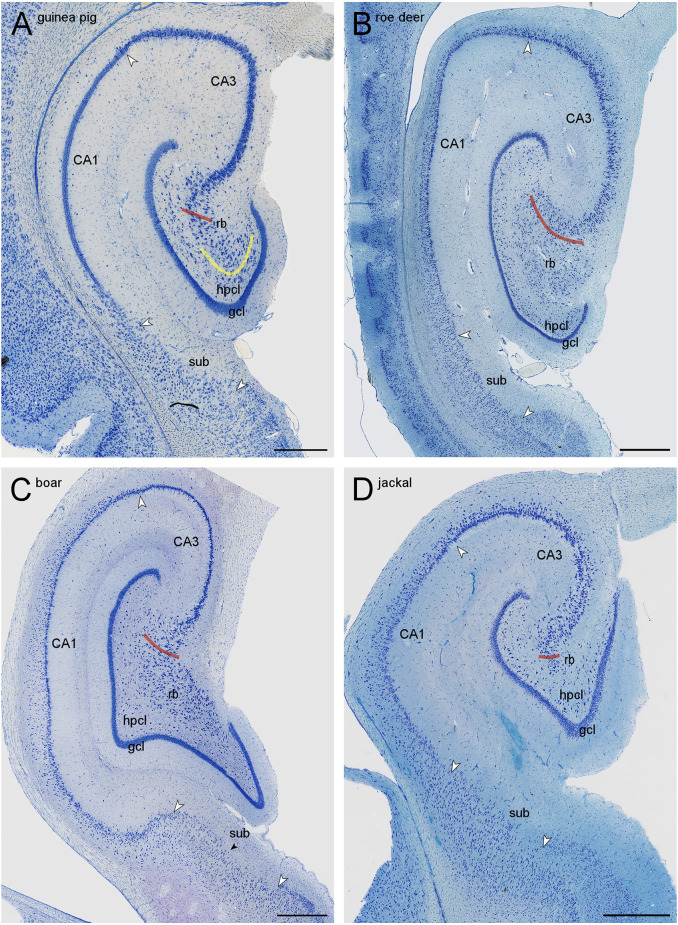
Mid-septotemporal sections of the hippocampal formations of guinea pig **(A)**, European roe deer **(B)**, wild boar **(C)**, and golden jackal **(D)**. Red lines mark the border of the CA3 reflected blade (rb) towards the remainder of CA3. Yellow lines mark the boundary between the reflected blade and the hilar polymorphic cell layer (hpcl) in species in which they partially merge. Large white arrowheads mark regional borders. The small black arrow in **(C)** marks the approximate boundary between the proximal subiculum (or prosubiculum) and the distal subiculum. In all species, the size of the reflected blade decreases towards the temporal pole of the hippocampus. The decrease is, in our species sample, most pronounced in the jackal, and only little of the reflected blade remains in the mid-septotemporal jackal hippocampus. Scale bars: **(A)** 0.5 mm; **(B,C)** and **(D)** 1 mm.

The **granule cell layer** forms a compact band of neurons (Figures 1, 2) that are morphologically distinct from the sparse neurons in the adjoining subgranular or fusiform layer (visible in guinea pig, Figure 2A) and of the hilar polymorphic cell layer.

The **hilar neuron population** does include neurons contained within the hilar polymorphic cell layer (hpcl, Figures 1A,B) including the subgranular layer and pyramidal basket cells scattered along the deep boundary of the granule cell layer. In all species of this sample, the polymorphic cell layer was separated from the reflected blade by a continuation of stratum radiatum that inserts itself between the reflected blade and the polymorphic cell layer (Figures 1, 2). In rock mouse, rabbit, and guinea pig, the proximal tip of the reflected blade merges with the polymorphic cell layer beneath the infrapyramidal blade of the granule cell layer. At this point, a short line continuing the curvature of the stratum radiatum was used to separate the reflected blade from the polymorphic cell layer (Figures 1A,E, 2A). This line usually also separated neurons with different somal morphologies and apparent packing densities.

The border of the **reflected blade** towards the remainder of CA3 was first marked at low magnification based on the distinct reflection of the cell layer and subsequently refined, at higher magnification, based on the orientation of the apical dendrites of the reflected blade neurons. Occasionally, pyramidal cells in a very short distal segment of the reflected blade markedly pointed their apical dendrites towards the neuropil layers of CA3 and away from the tip of the granule cell layer. If such a segment was present, these cells were included with CA3, and the border between the reflected blade and the remainder of CA3 did not necessarily coincide with the exact point of flexure (e.g., Figure 1A). Apical dendrites of reflected blade pyramidal cells mostly pointed towards the granule cell layer (Figure 1C2). In all species in this study, the reflected blade gradually decreases in size along the septotemporal axis, and it is not present in the very temporal hippocampus.

**CA3 pyramidal cells** appear much larger and less densely packed than those in the adjacent superficial CA1 pyramidal cell layer. Typically, the transition is fairly abrupt, with CA3 pyramids being replaced by CA1 pyramids within the width of a few cell bodies. When the mossy fiber zone was visible also in Giemsa-stained sections (Figure 1D), the point of transition from large CA3 to small CA1 pyramidal cells coincided with the distal tip of the mossy fiber zone. In addition, dense calbindin positive fibers marked the mossy fiber zone in rock mouse, guinea pig, rabbit, and jackal.

**CA2** was originally defined by Lorente de Nó (1934) as a small field distal to the end of the mossy fibers. Lein et al. (2005) redefined CA2 based on the expression of markers like Purkinje cell protein 4 (PCP4) as a small field largely proximal to the end of the mossy fibers. Both definitions are in current use (Kohara et al., 2014; San Antonio et al., 2014; Dudek et al., 2016; Lothmann et al., 2021). In the present material, we could not consistently define a distinct histoarchitectural field that may correspond to either of these definitions. Depending on which definition for CA2 is used, CA2 cells are here included in either CA1 or CA3.

The border between the **CA1 pyramidal cell layer** and the subicular cell layer is marked by the end of the stratum oriens at depth and the end of the compact superficial band of CA1 pyramidal cells. Also, cells in the subicular cell layer were morphologically more heterogeneous than in the adjacent CA1. Calbindin positive superficial CA1 pyramidal cells are found throughout the transverse and septotemporal axis in rock mouse and jackal, but not in the other species. When present, the loss of calbindin positive superficial pyramidal cells coincided with the cytoarchitectural changes that marked the beginning of the subiculum. Parvalbumin positive cells are more frequent and morphologically more diverse in the proximal subiculum than in adjacent CA1. Also, parvalbumin positive cells and neuropil are distributed more evenly in the subicular cell layer than in the adjoining CA1. These markers were helpful to confirm the border between the subiculum and a broad CA1 pyramidal cell layer in jackal, wild boar, roe deer, and red deer, in which the cytoarchitectural changes at the border are less pronounced than in rock mouse, guinea pig, or rabbit.

Throughout most of its septotemporal extent, the **subicular cell layer** borders the presubiculum. Changes in cytoarchitecture are marked in particular in presubicular layers II and V, and presubicular cell densities appear higher and somata smaller throughout all layers. Occasionally, small, isolated islands of presubicular layer II cells are scattered into the subicular plexiform layer. These cells were not counted. Temporally, the subicular cell layer borders the modified entorhinal cortex. The appearance of a lamina dissecans in the latter defined the border between the regions.

Proximal and distal subdivisions have been identified within the subiculum (e.g., Slomianka and Geneser, 1991; Honda and Ishizuka, 2015; Ishihara and Fukuda, 2016; Honda and Shibata, 2017; Cembrowski et al., 2018a, b; Ishihara et al., 2020). Based on additional comparative (Ding, 2013), connective, and gene expression data (Ding et al., 2020), Ding and co-workers reinstated regional status and adopted the term prosubiculum for the proximal subiculum and subiculum proper for the distal subiculum. While these subdivisions are visible in rock mouse (Figure 1E) and boar (Figure 2C), histoarchitectural differences are less marked in other species, and a consistent definition of subicular subfields/regions would not be possible in all species.

### Data analysis

To visualize the differences in the relative size of the neuron populations, we used a correspondence analysis in R (package MADE4; Culhane et al., 2005), which is similar to a principal components analysis. The correspondence analysis eliminates large differences in absolute cell numbers and associated differences in variances between species by using weighted Euclidean distances. Number estimates from each animal were log transformed and scaled by subtracting the mean of all neuron populations of that animal from each individual estimate and dividing by the standard deviation of the estimates. All animals, therefore, have cell counts with a mean of 0 and a standard deviation of 1 across all populations, while relative differences between populations are preserved (for a brief and simple numerical example see van Dijk et al., 2016a). In addition, a MANOVA was performed in R to test for the dependence of relative cell numbers on species.

## Results

### Correspondence analysis

**Figure 3:**
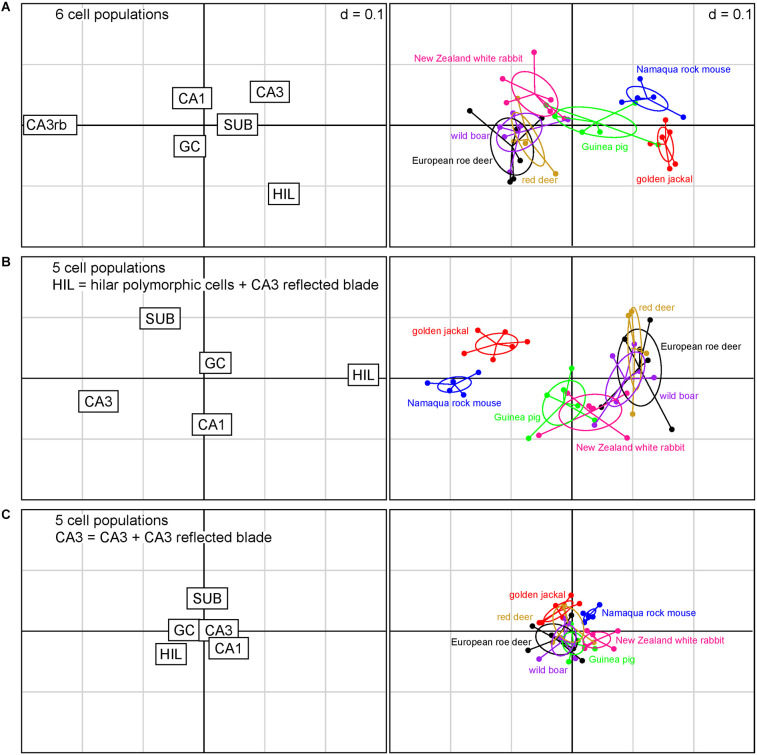
Correspondence analysis showing the relationships between species based on quantitative relations between hippocampal principal cell numbers. The plots of hippocampal principal cell populations (left) show their power to separate species (right). **(A)** The six cell populations are presented independently. Cell numbers in the reflected blade (CA3rb) and the hilar polymorphic layer (HIL) are the strongest differentiators between species. **(B)** Reflected blade cells were added to the hilar cell population. Hilar and CA3 populations are the strongest differentiators. **(C)** Reflected blade cells are added to the CA3 pyramidal cell population. Species coalesce into a dense cluster as the differentiating power of all cell populations decreases. In this Figure and in Figure 4; if the position of a species in the right graph is close to the position of a cell population in the left graph, the cell population will be large in that species. The position of a species will change as the relative sizes of the cell populations change the different models.

When all cell populations of interest are considered individually within the present sample of species (Figure 3A), reflected blade and hilar cell polymorphic population sizes were the strongest differentiators between species. Species tend to have either a large reflected blade population or a large hilar polymorphic population, but not both. CA3 pyramidal cells are weaker differentiators. Again, species may have a large population of CA3 or reflected blade pyramidal cells, but not both. Granule cells, CA1 pyramidal cells, and subicular neurons are yet weaker differentiators. Figure 3A captures about 80% of the variance in the data (1st factor, x-axis: 61.80%, 2nd factor, y-axis: 17.74%).

When reflected blade neurons and hilar polymorphic neurons are pooled into a hilar population (Figure 3B), the power of subicular, CA1, CA3, and hilar neurons to differentiate between the species increases. Also, rabbits and guinea pigs are differentiated from the other species by slightly larger CA1 pyramidal cell populations, while the jackal has a larger subicular population. Granule cells remain weak differentiators in this sample of species. Figure 3B captures about 82% of the variance in the data (1st factor, x-axis: 64.60%, 2nd factor, y-axis: 17.09%).

Finally, the hilar cell population was restricted to the neurons of the polymorphic cell layer, while reflected blade neurons were pooled with the remaining CA3 (Figure 3C). In absolute terms, the differentiating power of all populations is smaller than in the previous scenarios. The species contained in the present sample coalesce into a largely overlapping cluster. Hilar and subicular neurons retain some power to differentiate between species, while CA3 neurons almost completely lose theirs, being supplanted by CA1 and subicular neurons. Granule cell population size is once again a poor differentiator. Figure 3C captures about 90% of the variance in the data (1st factor, x-axis: 73.03%, 2nd factor, y-axis: 17.05%).

For all models, only the eigenvalues of the first factors exceeded the average of all factors.

As one should expect from the distribution of species data points, a MANOVA showed a strong dependency of relative cell numbers on species, but p values were 12 orders of magnitude larger for the scenario depicted in Figure 3C (*p* < 0.001) than for those in Figures 3A,B (*p* < 1 × 10^−15^).

**Figure 4:**
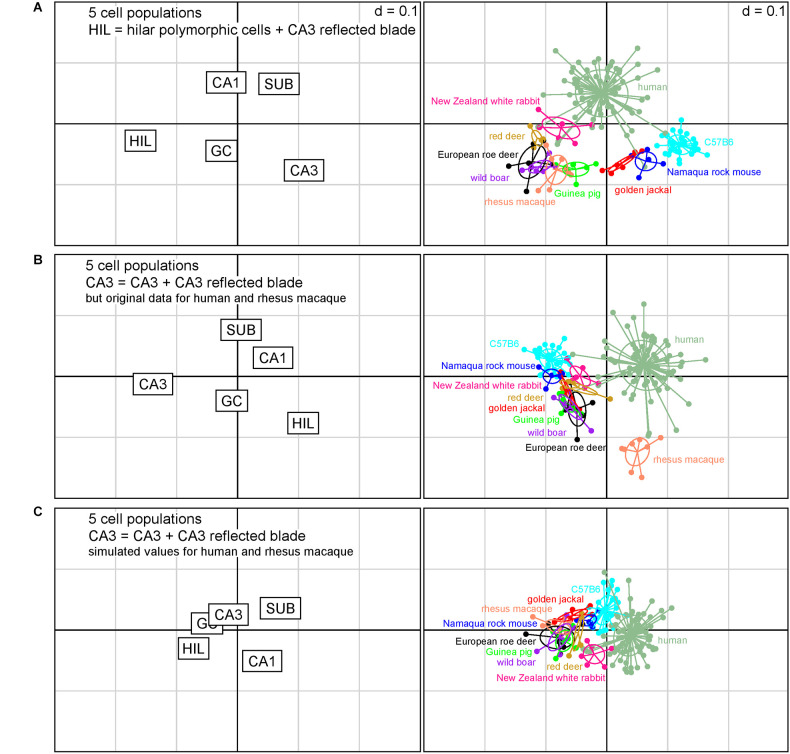
Correspondence analysis including in addition laboratory mice, rhesus macaque and humans. The plots of hippocampal principal cell populations (left) show their power to separate species (right). **(A)** Reflected blade neurons were pooled with hilar polymorphic cells. The seven species retain their relations also in the context of the three additional datasets from C57B6 mice, humans, and macaque monkeys. **(B)** Reflected blade neurons were pooled with the remainder of CA3 in the present species, while the reflected blade remained with a hilar cell population in humans and macaque monkeys. The seven species form a group with C57B6 mice, while separating from humans and macaque monkeys. **(C)** Simulated values for reflected blade and hilar polymorphic populations were calculated for humans and macaque monkeys based on the rabbit data and reflected blade numbers of all species were pooled with the remainder of CA3. All species fall into a cluster with relatively little differentiation between the species.

How does the assignment of cells affect the position of the present species relative to those that either does not have a reflected blade or in which reflected blade neurons cannot be easily distinguished from polymorphic hilar neurons based on histoarchitecture? To answer this question, we pooled the reflected blade neurons either with the polymorphic layer neurons (Figure 4A) or with CA3 (Figure 4B) and compared the present species with data from C57B6 mice, in which no reflected blade can be identified, and with humans and rhesus macaques, in which the reflected blade population were not differentiated from the hilar polymorphic cells.

When reflected blade cells were considered to belong to the hilar cell population (Figure 4A), guinea pig, wild boar and both deer formed a loose cluster with rhesus macaques. Rabbits were positioned between the cluster and humans, while Namaqua rock mice and jackal were located between the cluster and C57B6 mice. Figure 4A captures 87% of the variance in the data (1st factor, x-axis: 63.87%, 2nd factor, y-axis: 22.84%).

When reflected blade cells were considered to belong to the CA3 neuron population (Figure 4B), all species other than human and rhesus macaque, in which reflected blade cells still are part of the hilar population, form a cluster with little taxonomic differentiation. Figure 4B captures ~86% of the variance in the data (1st factor, x-axis: 64.22%, 2nd factor, y-axis: 21.99%).

Unfortunately, separate reflected blade and polymorphic cell data are not available for humans and macaques. To investigate what would happen if their reflected blade populations were assigned to CA3, we simulated reflected blade cell numbers by separating their hilar populations according to the ratio between reflected blade cells and polymorphic cells in the species that was closest to humans in Figure 4A, the rabbit. Similar to the analysis based on seven species (Figure 3C), the differentiating power of all cell populations decreased and species coalesced (Figure 4C). Taxonomic differentiation is poor, with C57B6 mice and rabbits filling the gap between humans and a cluster composed of all other species. Figure 4C captures 90% of the variance in the data (1st factor, x-axis: 66.40%, 2nd factor, y-axis: 23.19%).

For all models, only the eigenvalues of the first factors exceeded the average of all factors.

We tried to split hilar cell numbers into reflected blade and polymorphic populations based on the relative sizes of proximal (reflected blade) to distal CA3 pyramidal cell numbers reported by Jabès et al. (2011) in rhesus macaques. Using this approach, we obtained negative cell numbers for the human hilar polymorphic population, i.e., relative to CA3, even the combined reflected blade and polymorphic cell numbers are smaller in humans than reflected blade cell numbers alone in rhesus macaques.

Even though C57B6 mice do not show a reflected blade, we also, for the sake of completeness, divided their hilar population according to the proportions between polymorphic and reflected blade cells seen in rock mice. This resulted in a larger separation of guinea pig and jackal from other species (as compared to Figure 4A, not illustrated) when all six populations were considered separately, and in a separation of the C57B6-rabbit-human cluster from the other species, when reflected blade cells were pooled with CA3 (not illustrated). Lastly, there will always be an observer bias in the definition of population boundaries. To test the robustness of the observations, we cross-reassigned 10% of the populations forming “difficult” borders (CA1 and subiculum in wild boar and CA3 and reflected blade in jackal, guinea pig, and rock mice) to the bordering population. The value of 10% was chosen because it exceeds, in our opinion, any differences that could possibly result from differences in observer bias. The reassignment of cells from one to another population did of course alter the precise position of a species, but the changes were too small to impact the overall outcomes described above.

### The Golgi-stained reflected blade in the rabbit

**Figure 5:**
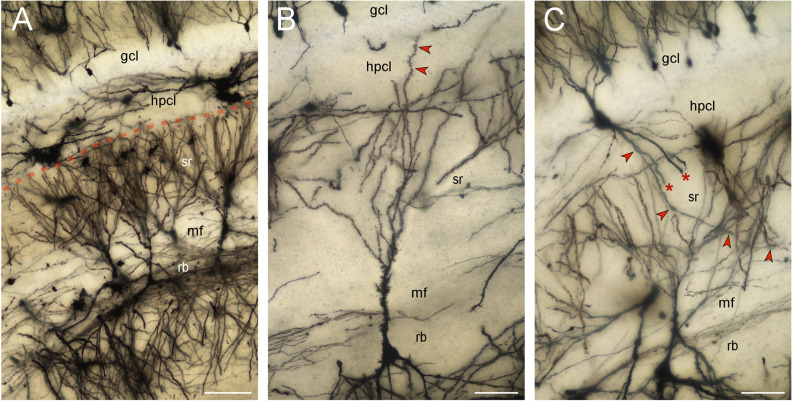
Golgi-stained reflected blade. Minimum density projections of image stacks. Out-of-focus elements of some planes were removed before the stacks were merged. **(A)** The vast majority of the apical dendrites of pyramidal cells located in the reflected blade (rb) respect the boundary (red stippled line) between the hilar polymorphic cell layer (hplc) and the extension of the CA3 stratum radiatum (sr) beneath the suprapyramidal blades of the granule cell layer (gcl) and hilar polymorphic cell layer (hplc). Scalebar: 100 μm. **(B)** A rare pyramidal cell extending one of its apical dendrites (red arrow heads) into the hilar polymorphic cell layer (hplc). The dendrite stops at the base of the granule cell layer (gcl). Scalebar: 50 μm. **(C)** A likely inhibitory interneuron with smooth or beaded dendrites extends an apical tuft of dendrites into the granule cell layer (gcl) and three large basal dendrites into the stratum radiatum (sr). Two of the basal dendrites are truncated (asterisk). The third one (red arrowheads) can be followed to the mossy fiber zone (mf). Scalebar: 50 μm.

CA3 pyramidal neurons did extend apical dendrites into the hippocampal stratum lacunosum-moleculare. Reflected blade CA3 pyramids (a few hundred well-impregnated cells) belonged exclusively to the non-apical type described previously, i.e., their apical dendrites did not reach the stratum lacunosum-moleculare nor did the apical dendrites turn into the directions of the hippocampus (Figure 5A). Instead apical dendrites fan out within the extension of the stratum radiatum below the supra- and infrapyramidal blades of the dentate gyrus. The vast majority of the terminal branches of the apical dendrites stop at the border between the stratum radiatum and the hilar polymorphic cell layer. Only very rarely was an apical dendrite traced into the hilar polymorphic cell layer (Figure 5B). They never extended beyond the base of the granule cell layer. A few neurons, located within the hilar polymorphic cell layer or the reflected blade, with beaded or smooth dendrites, i.e., most likely inhibitory interneurons, did cross the dentate/CA3 boundary (Figure 5C). A few pyramidal cells located at the boundary between CA3 and the reflected blade of CA3 did extend one apical dendrite towards the hippocampus whereas another apical dendrite was branching locally like typically reflected blade cells. Note that the Golgi-stain may not provide a complete or representative quantitative survey of the cell types.

### Short notes on the distribution of calretinin

C57B6 mouse sections, which were run as control with each staining batch of the other species, showed the staining pattern that has been described in the literature (Liu et al., 1996; Blasco-Ibáñez and Freund, 1997; Fujise et al., 1998). In all other species, we did observe strong calretinin-like (CR+) staining in cortical and hippocampal interneurons and a differentiated CR+ neuropil staining pattern in some cortical and hippocampal areas. Even though the presence or absence of CR+ stained elements did not seem to relate to postmortem delay (see “Animal and tissue preparation”), we cannot exclude altered staining patterns.

**Figure 6:**
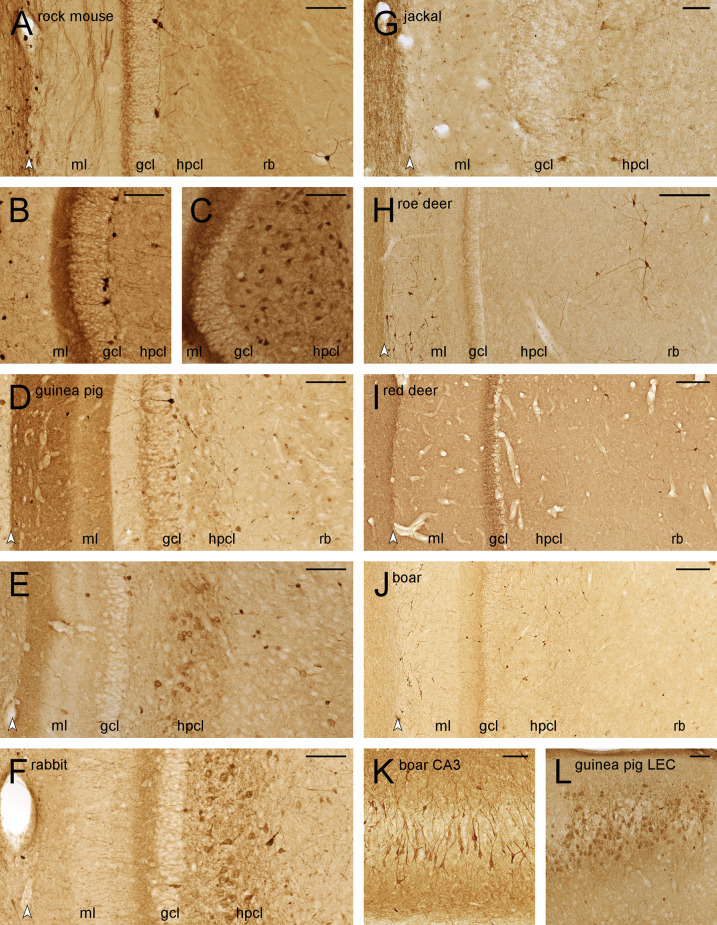
Calretinin-like immunoreactivity in the dentate gyrus. Septal **(A)**, temporal **(B)**, and extreme temporal **(C)** dentate gyrus of the rock mouse. While CR+ interneurons are found throughout the septotemporal axis of the dentate gyrus, frequent CR+ cells in the hilar polymorphic layer (hplc) are only seen at the temporal pole. Septal **(D)** and temporal **(E)** dentate gyrus of the guinea pig. Frequent CR+ are only found in the far temporal dentate gyrus. **(F)** Frequent CR+ cells are seen in the hplc of the temporal dentate gyrus of the rabbit. In the jackal **(G)**, roe deer **(H)**, red deer **(I)**, and wild boar **(J)** dentate gyrus, only interneurons are CR+. They are rare in the hplc and frequent in the reflected blade (rb) of both deer, while the pattern is reversed in wild boar. **(K)** A subpopulation of wild boar CA3 pyramidal cells is CR+. **(L)** CR+ cells are frequent in the cell island of the lateral entorhinal cortex (LEC) of the guinea pig. White arrowheads mark the borders between the hippocampal and dentate molecular layers. Abbreviations: ml, molecular layer; gcl, granule cell layer; hplc, hilar polymorphic layer; rb, reflected blade of CA3. Scale bars: **(A–G,K,** and **L)** 100 μm; **(H–J)** 250 μm.

CR+ staining was intended to further differentiate between the mossy cells and CA3 reflected blade cells. CR+ cells, possibly representing mossy cells, were only seen at the temporal extreme of the rock mouse (Figures 6A–C) and wild boar. CR+ mossy cells were absent from the septal and mid-septotemporal hilar polymorphic cell layer of guinea pig (Figure 6D) and rabbit, but present temporally (Figures 6E,F). CR+ cells with the morphology of mossy cells were absent from jackal (Figure 6G), roe deer (Figure 6H), red deer (Figure 6I), and aside from the temporal pole, also from wild boar (Figure 6J). The presence of CR+ mossy cells correlated well with the presence of CR+ staining in the commissural-associational zone of the dentate molecular layer, which was present and strongest temporally in rock mouse (Figures 6B,C), and weaker in guinea pig (Figure 6E), rabbit (Figure 6F), and wild boar (Figure 6J).

In deer and wild boar, CR+ interneurons were differentially distributed to the hilar polymorphic layer (very few in roe deer and red deer, frequent in wild boar) or the reflected blade (frequent in both deer, very few in wild boar).

CR+ deep CA3 pyramidal cells were observed in the temporal hippocampus of rabbit and deer, and more frequently and stronger stained in wild boar (Figure 6K). In all three species, staining was strongest in distal CA3, declining to very few stained cells proximally and none that could be identified as a pyramidal cell in the reflected blade (Figure 6J). Finally, a small cluster of CR+ deep pyramidal cells was also found at the transition from CA3 to CA1 in guinea pigs.

Small CR+ cells (presumably Cajal-Retzius cells) were observed in variable numbers in the outer dentate molecular layer of all species, but they were unusually large and frequent in roe deer (Figure 6H) and wild boar (Figure 6J). In the guinea pig, we also observed fairly strong CR+ staining in the medial and lateral performant pathways terminal fields in the dentate molecular layer (Figures 6D,E; Murakawa and Kosaka, 1999). Corresponding CR+ neurons were seen in layer II of the guinea pig medial and lateral entorhinal cortex (Figure 6L), but not in other species.

CR+ staining has also been used as a marker for young granule cells formed during adult hippocampal neurogenesis in laboratory mice (Brandt et al., 2003). Even though we previously found large numbers of newly born neurons in adjacent sections of these rock mice (Cavegn et al., 2013), they are not CR+ (Figure 6A). They were also absent from rabbit and deer. CR+ deep granule cells were seen in guinea pig (Figure 6D), jackal, and wild boar. Their variable apparent numbers may reflect the age of the animals and/or species-specific levels of adult neurogenesis or CR expression windows.

## Discussion

One question that we sought to answer was how the assignment of a single, small cell population, that of the CA3 reflected blade, impacts quantitative relations between hippocampal principal cell populations and their taxonomic assessment. For the species sample presented in this work, the impact is massive. The analysis of the reflected blade cells as a separate population or their assignment to a hilar cell population resulted in the ability to differentiate between species and taxonomic groups. The differentiation power of all cell populations decreased strongly, and taxonomic scatter imploded when reflected blade cells were pooled with the remainder of CA3. Moreover, also primates coalesced onto the cluster of other species when their simulated reflected blade cell populations were pooled with the remainder of CA3. This even attenuated the separation of humans from other species that was based on a large CA1 volume (Stephan, 1983) and cell population (Seress, 1988). Joining CA3 and reflected blade populations seems to partially normalize the quantitative makeup of the principal hippocampal cell populations across species.

No previous studies of hippocampal principal cell numbers, in which reflected blade neurons were resolved, have been performed. Only the study of Jabès et al. (2011) reported proximal CA3 cell number in the rhesus monkey, which, according to their illustrations, corresponds to the reflected blade as defined in our work. This report did unfortunately not provide the number of dentate polymorphic cells. Some support for our observations can be derived from two re-analyses (Vanier et al., 2019; Schilder et al., 2020) of a large dataset on the volumes of primate hippocampal components (Frahm and Zilles, 1994). Although using different frames of reference, both studies found a larger CA3 and smaller hilus, including the reflected blade, in humans as compared to most other primates. Simulated human polymorphic cell number became negative when we used the relative size of proximal CA3 in the rhesus monkey to estimate their number. This could be explained if the smaller volume of the human hilus is also due to a smaller reflected blade cell number. Our CA3 cell numbers would predict the accompanying increase in the volume of CA3, as a smaller reflected blade cell number suggests a larger CA3 cell number. These observations were made using different analytical approaches—phylogenetic generalized least squares and principal component analyses (Schilder et al., 2020), phylogenetic analysis of covariance (Vanier et al., 2019), and the phylogenetically blind correspondence analysis (present results and van Dijk et al., 2016a). The similarity of the observations despite different analytical approaches suggests some robustness of the observations. Interestingly, Schilder et al. (2020) interpreted the smaller volume of the human hilus as a reversion to an ancestral state. Cytoarchitecturally, the reflected blade appears quite small or is absent in prosimians (Stephan, 1983; Frahm and Zilles, 1994; Gary et al., 2019; and our own unpublished observations in gray mouse lemurs and black-and-white ruffed lemur).

The variable presence of calretinin in the cell populations described here is in agreement with the species variability in the distribution of this marker that has been noted for both the hippocampus (Murakawa and Kosaka, 1999) and other cortical areas (Hof et al., 1999; Hof and Sherwood, 2005). CR+ mossy cells are not a reliable marker that separates the hilar polymorphic cell layer from the CA3 reflected blade in species, in which they cannot be separated by other means. Instead, CR+ interneurons were differentially distributed to the reflected blade and polymorphic cell layers, but only in both deer and wild boar. The observation of CR+ CA3 pyramidal cells in deer and wild boar adds to previous observations in Proechimys (Fabene et al., 2001) and feliform and caniform carnivores (Pillay et al., 2021). The absence of CR+ CA3 pyramidal cells in the caniform jackal, together with the differential and species-specific distributions in interneurons, mossy cells, and neuroblasts, underline the high degree of species variability that is shown by this calcium-binding protein.

Critical for the evaluation of the functional and taxonomic importance of our findings is the validity of the decision to treat cell populations as separate entities or to assign them to one or another of the principal cell populations. The continuation of stratum radiatum between reflected blade cells and hilar polymorphic cells in our sample allowed their reliable separation from hilar polymorphic cells, but this is not the case in other species. In sengi (Slomianka et al., 2013), marmoset monkey (van Dijk et al., 2016a) or other primates (West and Gundersen, 1990; Keuker et al., 2003), reliably separating the reflected blade from hilar polymorphic cells is far more difficult than separating the reflected blade from the remainder of CA3. Although it is more convenient to pool reflected blade and hilar polymorphic cells, one must ask if differences between reflected blade cells and those in the remainder of CA3 pyramidal cells justify this partition. The reflected blade is a little less compact than the remainder of CA3 in rock mice, guinea pigs, and rabbits. But this is also the case in proximal CA3 of rodents without a recognizable reflected blade. Other than the reflection, cytoarchitectural differences between these species are small. However, in jackal, red deer, roe deer, and wild boar, the reflected blade consists of a widely dispersed cell population with little resemblance to the compact cell layer found in the remainder of CA3. This appearance separates the reflected blade of the jackal, deer, and wild boar from that of rock mice, guinea pig, and rabbit and from proximal CA3 in laboratory mice and rats. A characteristic feature of proximal CA3 pyramidal cells in laboratory rats and mice is their dendrites avoiding the hilar region but turning towards and branching within CA3 neuropil layers (Amaral, 1978). Instead, reflected blade neurons can extend their dendrites into the hilar region and even into the dentate molecular layer in primates, sheep, and pigs (Lim et al., 1997b; Buckmaster and Amaral, 2001; Buckmaster, 2005; Blackstad et al., 2022). Our observations in the Golgi-stained rabbit hippocampus also show that reflected blade pyramids differ from those in the remainder of CA3 in that they are all non-apical pyramidal cells, without an apparent possibility of direct entorhinal input. Although Golgi’s original illustration of these cells in rabbits appears quite schematic (Golgi, 2001), they agree well with our observations.

A further argument to treat the reflected blade as a separate population or as part of a hilar population is that species differences that result from these scenarios do reflect their phylogenetic relations. Rodents did form a cluster separated from other phylogenetic groups in our previous study (van Dijk et al., 2016a) and the sample of artiodactyla (wild boar, roe deer, and red deer) form a very tight group in the present study. Quantitative relations between hippocampal subfields change with phylogeny and can be related to changes in function (Todorov et al., 2019; Vanier et al., 2019; Schilder et al., 2020). One should therefore expect phylogenetically close species to cluster in quantitative analyses.

Beyond these arguments, the case for a functional separation between reflected blade cells and the remainder of CA3 rested largely on studies of the proximal part of CA3 in laboratory rodents. A more conclusive answer to the question of the assignment will depend on further information on the anatomical, genetic, and functional characteristics of the reflected blade. Separating the reflected blade from the remainder of CA3 does provide the resulting cell populations with a surprisingly high differentiating power. If this is an indicator of their importance in the functional differentiation of the hippocampal region, gaining further information should be well worth the effort. But one must admit that the alternative is hardly less interesting. If the reflected blade is pooled with the remainder of CA3, species coalesce into a cluster in which taxonomic groups overlap to a high degree. If smaller quantitative differences translate to smaller functional differences, this would mean a high degree of functional conservation across species—if not in extant species, then as a quantitative archetype that formed the basis of the anatomical and functional differentiation in the hippocampus that we find today.

## Data Availability Statement

The original contributions presented in the study are included in the article, further inquiries can be directed to the corresponding author.

## Ethics Statement

Ethical review and approval was not required for the animal study because all material was collected post mortem and, according to Swiss law, the collection did not require ethics committee approval or other permits. Applicable laws or permits held by donors of the material are listed in Table 1 of the manuscript.

## Author Contributions

JM: material collection, processing and analysis, revisions of the manuscript. IA: collection and processing of material, revisions of the manuscript. LV and DL: collection of material, revisions of the manuscript. DW: revisions of the manuscript. LS: analysis of the material, drafting and revisions of the manuscript. All authors contributed to the article and approved the submitted version.
